# Aeromonas: a book review

**DOI:** 10.3389/fmicb.2015.00106

**Published:** 2015-02-12

**Authors:** Télesphore Sime-Ngando

**Affiliations:** LMGE, Laboratoire Microorganismes: Génome et Environnement, UMR Centre National de la Recherche 6023, Clermont Université Blaise PascalAubière, France

**Keywords:** Aeromonas, taxonomy, pathogenicity, beneficial associations, antigen structures, virulence mechanisms, molecular diagnostics, Aeromonas in food and water

“*Aeromonas*” (Graf, 2015) is a new coming reference hardback and ebook that provides an overview on taxonomy, ecology, and pathogenicity of the genus *Aeromonas*, a group of bacterial species in the gamma subclass of the Proteobacteria (i.e., Gammaproteobacteria). *Aeromonas* is a Gram-negative, motile, and facultative anaerobic bacterium. Some of the members are associated to infections in humans, fish, and other animals, and are widespread in aquatic environments (Austin et al., 1996). The book consists of nine chapters that integrates contributions from 13 expert-scientists, under the editorship of Joerg Graf (University of Connecticut, USA) who attributes its conception and the groundwork to Amy HORNEMAN (University of Maryland, USA). The overview of the specific topics is broad while providing a concise and comprehensive text for everyone involved with *Aeromonas* research.

The first chapter—*Introduction and overview*—describes the roots of research and the increasing interest of scientists in the *Aeromonas* field, and summarizes the chapter contents. This concisely represents an up-to-date state of the art of the covered topics that include taxonomy, fish pathogens, infections in humans, beneficial associations, antigen structures, virulence mechanisms, molecular diagnostics, and *Aeromonas* in food and water.

Chapter two (*Taxonomy*) provides a more or less chronological history of the *Aeromonas* taxonomy and its major changes since 1891 (i.e., date of the first reference to *Aeromonas*-like organism in the literature) with technological advances, from DNA–DNA hybridization coupled with physiological and biotechnological characterization to fingertprinting, gene sequencing, and whole-genome sequencing analyses. This has resulted in a great expansion of the genus *Aeromonas* although several taxonomic controversies persist for reliable species identification. General recommendations to meet such identification is provided by the author, which is a good point of this chapter.

Chapters three (*Aeromonas fish pathogens*) and four (*Aeromonas infections in humans*) are dedicated to *Aeromonas* diseases. The first diseases of fish are perhaps known from *Aeromonas* pathogens, including the two oldest known fish pathogens: the motile *A. hydrophila* and the non-motile *A. salmonicida*. By reviewing data on taxonomy, diagnosis, ecology, virulence factors, and disease control, the author of chapter three provides evidence that the number of *Aeromonas* fish pathogen species increases with increasing research efforts, although major gaps persist, primarily on their ecology. Chapter four weakens the controversial role of *Aeromonas* in causing diseases in humans. The authors further extend the *Aeromonas* disease range in humans, from gastroenteritis to other infections (nosocomial, bacteremia, pneumonias, and peritonitis).

Chapter five (*Aeromonas, a multifaceted microbe: beneficial associations with animals*) is short but original compared to other single-genus book dedicated to pathogens, because it provides examples of benign or even benefial associations of *Aeromonas*-animal pairings, primarily the case for the digestive tract symbiosis between *A. veronii* and medicinal leeches, a fascinating topic.

In chapter six (*Aeromonas antigenic structures*), an overview of the plethora of surface structures that facilitate interactions between *Aeromonas* and diverse hosts and evironments are elegantly given to the readers, including flagella, pili and fimbriae, lipopolysaccharide (LPS), outer membrane proteins, capsule, and the S-layer. The chapter is specifically focused on the recent findings on those external antigenic structures thought to have roles in *Aeromonas* pathogenesis, which offer targets for the development of vaccines.

Chapter seven (*New developments on the virulence mechanisms of Aeromonas hydrophila*) covers new virulence factors and the related molecular requirements for causing diseases, focusing primarily on the type III and IV secretion systems and the role of quorum-sensing systems in modulating *Aeromonas* virulence. Emphasis is also given to the role of secreted effector proteins and other virulence factors.

Chapter eight (*Molecular diagnostics by genetic methods*) deals with genetic methods used for classification and molecular diagnostics of *Aeromonas* species, including strategies designed for identifying strains at the species level from both pure cultures and environmental samples. Due to its complex historical taxonomy, *Aeromonas* appeared as one of the best examples to illustrate applications of molecular biology techniques in microbiology, integrating advantages and disadvantages, performance and limitations of molecular diagnostic microbial methods.

The nine and last chapter (*Occurrence and virulence potential of Aeromonas in food and water*) presents results from classical culture-dependent and 16S rRNA gene studies in the context of the ecology, prevalence and environmental factors, primarily temperature, influencing the proliferation and regrowth of *Aeromonas* in water, biofilm, and food.

Overall, I found the book timely because, as stated in chapter six, “since the previous book on Aeromonas over fifteen years ago there have been a number of aeromonad genomes sequenced that have been a great asset in helping laboratories to unravel the biology of this enigmatic pathogen.” Chapters are well-written, informative, well-referenced, and represent a valuable resource for scientist community in the Aeromonas field, including insights into relevant diagnostic methodologies. I particularly like the inclusion of chapter five on the beneficial Aeromonas-animal asssociations that opens the window to the evolutionary processes and genomic innovations which are the mother of the complex and generalized biological interactions in the modern world (Sime-Ngando et al., in press). Although, general concepts (interactomics, molecular dialogue, maturation of immunity system, host manipulation by parasites, etc.) related to biological associations, symbioses, and microbiota, including pathogenic and parastitic associations, are not developed (Cézilly et al., 2014; Llewellyn et al., 2014; Biron et al., 2015), likely because such concepts have not yet being fully applied to the Aeromonas research. This is also partly because the book lacks a chapter on general conclusions and perspectives that could have summarize its content with targeted taking-home messages, and projected the Aeromonas research field in the increasing ongoing scientific developments on biological associations. This is a compelling challenge facing contemporary biology and health ecology because in the natural world no organism exists in absolute isolation and thus every organism must interact with the environment and other organisms, pehaps largely outnumbering those organisms with typically “free-living” lifestyles upon which the bulk of our knowledge in biology is known (Sime-Ngando et al., in press).

## Conflict of interest statement

The author declares that the research was conducted in the absence of any commercial or financial relationships that could be construed as a potential conflict of interest.
